# Age-Related Differences in Vancomycin-Associated Nephrotoxicity and Efficacy in Methicillin-Resistant *Staphylococcus aureus* Infection: A Comparative Study between Elderly and Adult Patients

**DOI:** 10.3390/antibiotics13040324

**Published:** 2024-04-03

**Authors:** Lin Xi, Shanshan Li, Mengting Chen, Xiaolan Huang, Nanyang Li, Nanye Chen, Hailan Wu, Qiyu Bian, Xingchen Bian, Xin Li, Minjie Yang, Xiaoyu Liang, Jufang Wu, Beining Guo, Yaxin Fan, Jing Zhang

**Affiliations:** 1 Institute of Antibiotics, Huashan Hospital, Fudan University, Shanghai 200040, China; 22211220011@m.fudan.edu.cn (L.X.); chenmengting@huashan.org.cn (M.C.); 22111220017@m.fudan.edu.cn (X.H.); wuhailan@huashan.org.cn (H.W.); lixin@huashan.org.cn (X.L.); 13111220011@fudan.edu.cn (M.Y.); victorliang0@gmail.com (X.L.); wujufang@huashan.org.cn (J.W.); guobeining@huashan.org.cn (B.G.); 2 Key Laboratory of Clinical Pharmacology of Antibiotics, National Population and Family Planning Commission, Shanghai 200040, China; 3 National Clinical Research Center for Aging and Medicine, Huashan Hospital, Fudan University, Shanghai 200040, China; 4 Huashan Worldwide Medical Center, Huashan Hospital, Fudan University, Shanghai 200040, China; lishanshan@huashan.org.cn; 5 Phase I Clinical Research Center, Huashan Hospital, Fudan University, Shanghai 200040, China; linanyang@huashan.org.cn (N.L.); 15000331507@163.com (N.C.); bianxingchen@huashan.org.cn (X.B.); 6 Division of Evolution, Infection and Genomics, School of Biological Sciences, Faculty of Biology, Medicine and Health, University of Manchester, Manchester M13 9PT, UK; qiyu.bian@postgrad.manchester.ac.uk

**Keywords:** vancomycin, nephrotoxicity, trough concentration, AUC_24_, elderly patients, adult patients

## Abstract

Elderly patients (age ≥ 65 years) are susceptible to methicillin-resistant *Staphylococcus aureus* (MRSA) infections, with potential for more adverse treatment outcomes or complications compared to younger adults (18–64 years). This study compared vancomycin-associated nephrotoxicity and efficacy in elderly and adult patients and investigated the correlation between vancomycin pharmacokinetic/pharmacodynamic (PK/PD) indices and clinical outcomes. A prospective study was conducted in 10 hospitals in Shanghai from October 2012 to November 2019. A total of 164 patients with MRSA infections were enrolled, including 83 elderly and 81 adult patients. Vancomycin therapeutic drug monitoring (TDM) was performed in all patients, indicating significantly higher vancomycin trough concentrations (C_trough_), 24-h area under the curve (AUC_24_) values, and AUC_24_/minimum inhibitory concentration (AUC_24_/MIC) values in elderly patients compared to adult patients. The incidence of vancomycin-associated nephrotoxicity was nearly three times higher in elderly patients (18.1% vs. 6.2%, *p* = 0.020), despite similar clinical and microbiological efficacy. Of particular importance, a C_trough_ > 20 mg/L was found as an independent factor of nephrotoxicity in elderly patients. Further analysis of patients with an estimated glomerular filtration rate (eGFR) > 60 mL/min/1.73 m^2^ also revealed that elderly patients had significantly higher vancomycin-related PK/PD indices and more nephrotoxicity than adult patients. In conclusion, elderly patients receiving vancomycin therapy face a higher risk of nephrotoxicity, which requires close vancomycin TDM, especially when the C_trough_ exceeds 20 mg/L.

## 1. Introduction

Elderly patients (age ≥ 65 years), often with diminished immunity and multiple comorbidities, are particularly susceptible to *Staphylococcus aureus* infection, a common cause of hospitalization with a rising incidence [1]. Notably, methicillin-resistant *S. aureus* (MRSA) bacteremia and pneumonia disproportionately affect elderly patients compared to younger adult patients (aged 18–64 years), with higher mortality rates [2].

Vancomycin, a first-line therapy for MRSA treatment, has been applied in clinical practice since the 1950s. It is a time-dependent antibiotic with an optimal pharmacokinetic/pharmacodynamic (PK/PD) index of the 24-h area under the curve/minimum inhibitory concentration (AUC_24_/MIC) [3]. The revised 2020 Infectious Diseases Society of America (IDSA) Guidelines [4] prioritize monitoring the AUC_24_/MIC ratio of 400–600 over a vancomycin trough concentration (C_trough_) of 15–20 mg/L for optimal clinical efficacy and patient safety. The 2020 Chinese evidence-based guideline for therapeutic drug monitoring of vancomycin [5], however, continues to recommend monitoring both the steady-state C_trough_ (10–20 mg/L for MRSA infections) and AUC_24_ (400–650 mg·h/L). Despite recommending therapeutic drug monitoring (TDM) of vancomycin in elderly patients, the supporting evidence in the guideline [5] is of low quality and lacks high-quality and specific literature.

A cohort study [6] demonstrated that applying a standardized dosing regimen in MRSA patients led to significantly higher C_trough_ and AUC_24_/MIC values in older patients (≥65 years) compared to younger adults (18–64 years), while the incidence of nephrotoxicity was similar between the two groups. However, it was well known that higher levels of both C_trough_ and AUC_24_ were strongly associated with nephrotoxicity risk [7,8], and further comparative analyses of two patient groups are necessary. Additionally, the study was retrospective, and the MIC was determined using two different methods, which may affect the calculation of the AUC_24_/MIC [9]. Hence, prospective clinical studies and harmonized methods for MIC determination are also needed to further validate these findings.

To evaluate the impact of age on vancomycin-related treatment efficacy and nephrotoxicity, we conducted a prospective clinical trial in elderly and younger adult patients. Additionally, an analysis of the factors influencing these clinical outcomes, including patient clinical characteristics and vancomycin PK/PD indices, was conducted to identify the corresponding risk factors in both groups.

## 2. Results

### 2.1. Patient Enrollment and Characteristics

The study enrolled 164 patients with MRSA infections, including 83 patients aged ≥65 years, with a median age of 80 years (interquartile range [IQR]: 75–85), and 81 patients aged 18–64 years, with a median age of 53 years (IQR: 40–60).

While initial creatinine levels were similar between older and adult patients, the two groups exhibited significant differences in initial and final eGFR, final creatinine, and creatinine change (Table 1). Elderly patients with MRSA infections exhibited significantly higher rates of comorbidities compared to adult patients, including cardiovascular disease (51.8% vs. 16.0%, *p* < 0.001), diabetes mellitus (26.5% vs. 6.2%, *p* < 0.001), stroke (43.4% vs. 21.0%, *p* = 0.002), and chronic obstructive pulmonary disease (COPD) (10.8% vs. 0.0%, *p* = 0.003). Furthermore, elderly patients were less likely to undergo surgery (26.5% vs. 66.7%, *p* < 0.001) and were more susceptible to pneumonia (77.1% vs. 60.5%, *p* = 0.022). However, adult patients were more prone to skin and soft tissue infections (18.5% vs. 3.6%, *p* = 0.002) and received treatment with quinolones more frequently (7.4% vs. 0.0%, *p* = 0.013).

### 2.2. Vancomycin Treatment and Clinical Outcomes

Despite receiving a lower daily vancomycin dose [1.4 (1.0, 2.0) g vs. 2.0 (1.8, 2.2) g, *p* < 0.001], elderly patients still exhibited significantly higher vancomycin C_trough_ [14.69 (10.58, 20.71) mg/L vs. 7.82 (4.52, 12.57) mg/L, *p* < 0.001], AUC_24_ [461.65 (353.01, 609.10) mg·h/L vs. 387.56 (283.53, 462.09) mg·h/L, *p* = 0.006], and AUC_24_/MIC [647.97 (461.65, 913.42) vs. 463.54 (330.09, 718.28), *p* = 0.007] values than adult patients (Table 1 and Figure S1 in Supplementary Materials). There was no difference in vancomycin peak concentrations (C_peak_) and MIC values between the two groups (Table 1 and Figure S2 in Supplementary Materials).

Clinical and microbiological success rates did not differ significantly between the two groups. However, the incidence of nephrotoxicity was significantly higher in the older group (18.1% vs. 6.2%, *p* = 0.02), with a nearly 3-fold increase (Table 1). In addition, 3 out of 15 patients in the elderly group experienced nephrotoxicity within 48 h, while 2 out of 5 patients in the adult group developed nephrotoxicity within the same timeframe.

### 2.3. Risk Factor for Nephrotoxicity

Risk factors for nephrotoxicity were analyzed in the older and adult groups. Among the older patients, 15 experienced nephrotoxicity following vancomycin therapy (Table 2). A univariate analysis identified the following factors associated with nephrotoxicity (*p* < 0.1): surgery, drainage tube, ICU admission, C_trough,_ C_peak_, and AUC_24_. In the subsequent multivariate logistic analysis, only a C_trough_ > 20 mg/L still had a significant association with nephrotoxicity (Table 3). 

In contrast, for the adult group, significant differences were observed in initial serum creatinine, initial eGFR, daily dose, C_trough_, C_peak,_ AUC_24_, and AUC_24_/MIC (Table 2), but the multivariate analysis did not reveal any significant associations between these factors and nephrotoxicity (Table S1 in Supplementary Materials).

### 2.4. Associations between Nephrotoxicity and Vancomycin Exposures

**Figure 1:**
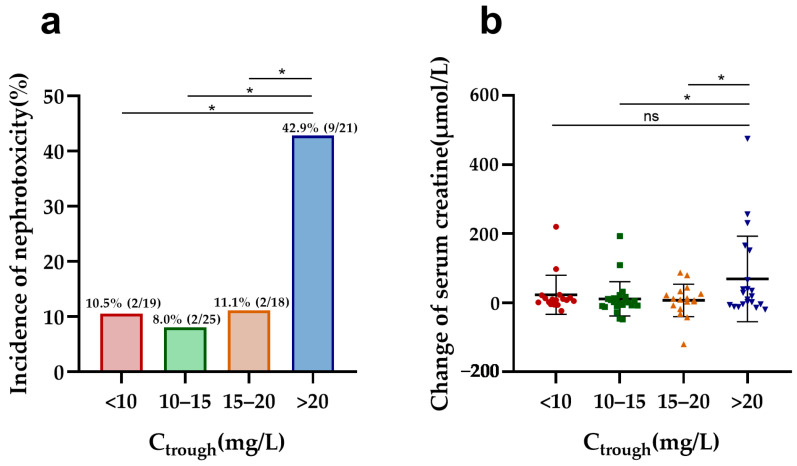
Nephrotoxicity and serum creatine changes in elderly patients by different C_trough_ groups: (**a**) comparison of the incidence of nephrotoxicity among different C_trough_ groups in elderly patients; (**b**) comparison of change in serum creatine levels during vancomycin treatment across different C_trough_ groups in elderly patients. * *p* < 0.5; ns: no significance.

**Figure 2:**
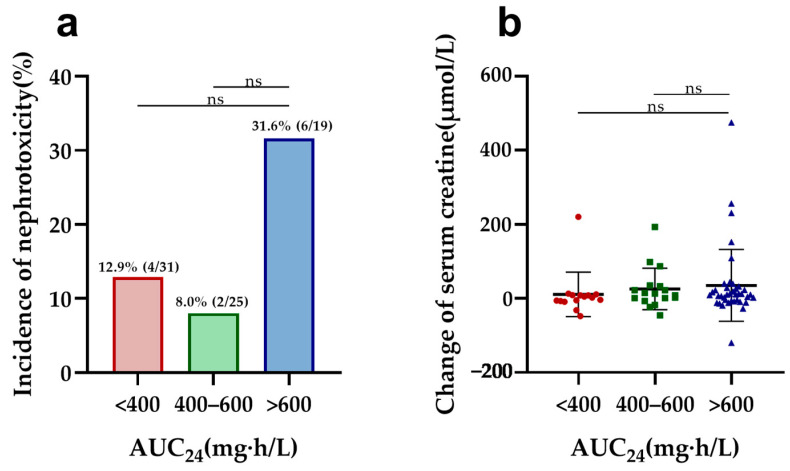
Nephrotoxicity and serum creatine changes in elderly patients by different AUC_24_ groups: (**a**) comparison of the incidence of nephrotoxicity among different AUC_24_ groups in elderly patients; (**b**) comparison of change in serum creatine levels during vancomycin treatment across different AUC_24_ groups in elderly patients. ns: no significance.

In vancomycin therapy, C_trough_ and AUC_24_, identified as markers of nephrotoxicity, were further evaluated in both elderly and adult patients. Elderly patients with a higher vancomycin C_trough_ (>20 mg/L) had a significantly increased risk of nephrotoxicity compared to other trough concentrations (C_trough_ < 10, 10–15, and 15–20 mg/L) and a more pronounced elevation in serum creatinine compared to those with C_trough_ values of 10–15 and 15–20 mg/L (Figure 1). Furthermore, C_trough_ was also found to be a good predictor of nephrotoxicity in elderly patients, with an AUC of 0.740 (95%CI, 0.588–0.891, *p* = 0.004) using the ROC analysis (Figure S3 in Supplementary Materials). The optimal cutoff value was 20.78 mg/L, with a sensitivity of 60.0% and a specificity of 83.8%. However, there was no difference in nephrotoxicity and creatine changes between the different AUC_24_ groups (Figure 2). Also, the cutoff values of the AUC_24_ were not significant for predicting nephrotoxicity in older patients (*p* > 0.05). 

While significant changes in nephrotoxicity and SCr rise were also observed among young adults, categorized by C_trough_ and AUC_24_, further analysis revealed limitations due to the overall low incidence of nephrotoxicity in this group. The small number of cases, combined with the fact that nearly all nephrotoxicity occurred in the high-exposure group (C_trough_ > 20 mg/L and AUC_24_ > 600 mg·h/L), restricted the ability to identify statistically significant associations in the multivariate analysis.

### 2.5. Associations between Nephrotoxicity and Renal Function

Considering age-related differences in renal function, patients with normal renal function were compared between the elderly and adult groups. Consistent with the findings in the entire cohort, the subgroup analysis of patients with an eGFR exceeding 60 mL/min/1.73 m^2^ yielded similar results. Elderly patients consistently exhibited significantly higher vancomycin C_trough_, AUC_24_, and AUC_24_/MIC values compared to adult patients. Remarkably, elderly patients with normal renal function exhibited a significantly higher incidence of nephrotoxicity compared to adult patients (15.4% vs. 2.7%, *p* = 0.007). Importantly, no significant differences in clinical and microbiological outcomes were observed between the two groups (Table 4). Overall, all data suggest that elderly patients, even with normal renal function, may remain more susceptible to vancomycin-induced nephrotoxicity compared to adult patients.

## 3. Discussion

In this study, we compared the efficacy and nephrotoxicity of vancomycin therapy in MRSA-infected patients between elderly (≥65 years) and adult patients (18–64 years). A notable finding was the significantly higher incidence of nephrotoxicity among elderly patients (18.1% vs. 6.2%), despite comparable clinical success and microbiological clearance rates. Previous studies have demonstrated that reduced albumin, a higher volume of distribution, decreased renal function, and a longer half-life in elderly patients can alter the PK/PD of vancomycin [10,11]. However, current vancomycin dosage and monitoring guidelines fail to consider elderly groups. Our findings underscore the need for age-tailored dosing strategies and the comprehensive monitoring of vancomycin therapy in elderly patients to minimize the risk of adverse events, particularly nephrotoxicity.

Currently, there is still a lack of cohort studies comparing vancomycin treatment in elderly and adult patients with MRSA infections. Only Yahav et al. has conducted such a comparison to date, who reported no significant differences in nephrotoxicity between elderly and adult patients. However, we observed a three-fold higher incidence of nephrotoxicity in elderly patients compared to adults. This discrepancy may be attributable to differences in nephrotoxicity assessment criteria: Kidney Disease: Improving Global Outcomes (KDIGO) in our study vs. Risk, Injury, Failure, Loss, End-Stage Kidney Disease (RIFLE) criteria in their study [12,13]. Additionally, baseline creatinine levels of both groups in Yahav’s study were higher than that of our study, which may affect the risk of nephrotoxicity occurrence.

In our study, elderly patients exhibited significantly higher vancomycin C_trough_, AUC_24_, and AUC_24_/MIC values compared to adult patients. Our multivariate logistic regression analysis indicated that a C_trough_ > 20 mg/L is an independent risk factor for nephrotoxicity, with an optimal cutoff of 20.78 mg/L for elderly patients based on ROC analysis. Moreover, elderly patients with a C_trough_ exceeding 20 mg/L exhibited a significant increase in nephrotoxicity incidence and serum creatinine changes compared to those with lower C_trough_ values. While a significant correlation exists between serum vancomycin C_trough_ and the incidence of vancomycin-associated nephrotoxicity [14], the specific C_trough_ threshold for increased risk remains inconsistent among elderly patients. Wang et al. suggested that a vancomycin C_trough_ ≥ 20 mg/L seems to indicate a poorer prognosis in patients ≥80 years [15], while Fukumori et al. proposed that nephrotoxicity risk increases with a C_trough_ in excess of 15 mg/L [3]. Dai et al. further recommended different C_trough_ thresholds based on renal function, with 21.5 mg/L and 16.5 mg/L associated with increased nephrotoxicity risk in chronic kidney disease at Stage 3a and 3b-5, respectively [16]. The C_trough_ threshold for vancomycin-induced nephrotoxicity in elderly patients may require further research for determination.

The 2020 IDSA Guideline and some reports [17,18] concluded that C_trough_ was a poor predictor of AUC_24_. However, our study revealed a correlation between C_trough_ and AUC_24_ in both groups, indicating that to some extent, C_trough_ can reflect the situation of AUC_24_ (Figure S4 in Supplementary Materials). In clinical practice, actively monitoring C_trough_ levels proves beneficial and effective for elderly patients at risk of nephrotoxicity, especially when C_trough_ is maintained above 20 mg/L.

During vancomycin treatment, close monitoring of serum creatinine levels is essential in elderly patients, as they exhibit a significantly higher rise in serum creatinine compared to adult patients. This increased susceptibility to vancomycin-induced nephrotoxicity is attributed to microanatomical structural changes in the aging kidney, including a reduction in the number of functional glomeruli due to an increased prevalence of nephrosclerosis, a compensatory hypertrophy of remaining nephrons [19], and a diminished kidney functional reserve compared to adult patients. Renal function is strongly related to vancomycin C_trough_ [20]. In elderly patients, lower empiric vancomycin doses and shorter duration are advised due to reduced clearance from enhanced tissue binding and overall decreased systemic and renal clearance [21]. Hence, vigilant monitoring of vancomycin levels and renal function is crucial for elderly patients. Additionally, considering that some patients may develop AKI within 48 h after vancomycin administration, it is preferable to initiate monitoring as early as possible (within 48 h).

Despite receiving a lower daily vancomycin dose, elderly patients still exhibited significantly higher vancomycin C_trough_, AUC_24_, and AUC_24_/MIC values compared to adult patients. The difference is likely attributed to complexities of renal function assessment in older patients, potentially influenced by factors such as sarcopenia [6] and hypoalbuminemia, which can impact vancomycin pharmacokinetics. These findings were consistent even in a subgroup analysis of patients with normal eGFRs (eGFR > 60 mL/min/1.73 m^2^), suggesting that creatinine-based GFR estimation in elderly patients may not accurately reflect their true renal function.

In addition to vancomycin-associated indices, ICU residence was associated with nephrotoxicity in elderly patients [22]. This increased susceptibility likely stems from altered vancomycin pharmacokinetics [23,24,25], sepsis, hemodynamic instability, contrast exposure, and concurrent nephrotoxic medication [26].

The limitations of this study are that patients with only one measured concentration were excluded from the AUC_24_ calculation, and no adjustment was made for prognostic factors such as Acute Physiology and Chronic Health Evaluation (APACHE) II score and frailty status. Future studies are needed to confirm the findings using larger sample sizes and more comprehensive data collection.

## 4. Materials and Methods

### 4.1. Study Design and Data Collection

This cohort was obtained from two prospective, multicenter, clinical observational studies conducted between October 2012 and November 2019 that were registered with the Chinese Clinical Trial Registry (Registration numbers: ChiCTR-OPC-16007920 and ChiCTR-OPC-17012567). The study was approved by the ethics committees of Fudan University-affiliated Huashan Hospital and various sub-centers. All participants signed informed consent before being enrolled in the study.

Patients were classified into elderly patients (≥65 years) and younger adult patients (18–64 years). Demographic information from patients, including gender, age, body mass index (BMI), renal function, comorbidities (such as cardiovascular diseases, diabetes mellitus, stroke, chronic obstructive pulmonary disease, autoimmune diseases, malignancy, hematological malignancy, etc.), types of medical intervention (venous catheters, tracheal cannula, tracheostomy, urinary catheters, drainage tubes, surgery, ICU admission, etc.), infection sites, duration of vancomycin treatment, vancomycin daily dosage, and concomitant use of other antibiotics were collected. The eGFR (estimated glomerular filtration rate) was calculated by the modification of diet in renal disease equation. The change in serum creatinine was determined by subtracting the serum creatinine measured either on the day before or on the initial day of vancomycin therapy from the maximum serum creatinine recorded during the course of therapy. These data were extracted from medical records and verified by two physicians.

### 4.2. Inclusion and Exclusion Criteria

Patients included in the study had bloodstream infections, pulmonary infections, central nervous system infections, skin and soft tissue infections, urinary tract infections, and other infections caused by MRSA, confirmed by clinical symptoms, physical signs, laboratory tests, and microbiological examinations. Patients were aged 18 years or older and treated with vancomycin for at least 5 days.

Individuals were excluded from this study if they had received effective anti-MRSA agents such as linezolid, norvancomycin, or daptomycin for more than 24 h within 72 h before inclusion, were colonized with MRSA, received less than 5 days of vancomycin treatment, underwent dialysis, were pregnant or lactating, or lacked a microbiological diagnosis of infection.

### 4.3. Microbiological Detection

Pathogens were identified using the Mérieux Vitek2 GP identification card (version number: 21342) and the VITEK 2 Compact system (BIOMÉRIEUX, Marcy l′Etoile, France). The MICs of vancomycin and oxacillin were determined using the agar dilution method according to the Clinical and Laboratory Standards Institutes (CLSI) standards [27,28], with ATCC29213 as the quality control strain. *S. aureus* with an oxacillin MIC of ≥4 mg/L was classified as MRSA.

### 4.4. Vancomycin TDM and PK/PD Analysis

Clinicians initiate vancomycin therapy based on the patient’s renal function. For patients with normal renal function, 15–20 mg/kg of vancomycin is administered based on actual body weight every 8–12 h as an intravenous infusion [29]. Vancomycin TDM and PK/PD analysis were conducted to enhance the efficacy and safety of treatment for all enrolled patients. For patients with normal kidney function, C_trough_ samples were collected 0–0.5 h before the 4th or 5th dose, and peak concentration samples were collected at 0.5–1 h after the end of vancomycin infusion. For patients with impaired or severe kidney function (i.e., eGFR < 30 mL/min/1.73 m^2^), serum C_trough_ and peak concentration samples were collected before and after the second vancomycin dose, respectively. If the patient’s dosage was adjusted, TDM could be repeated after 3–4 days of vancomycin therapy, depending on how the patient’s infection was responding to treatment.

Vancomycin concentrations were measured using chemiluminescent immunoassay or fluorescence polarization immunoassay, with a linear range of 3–100 mg/L. Quality control and external quality assurance samples were performed to monitor assay differences. The vancomycin AUC_24_ was calculated using Fan’s method [30]. Specifically, a one-compartment model in Phoenix WinNonlin 8.0 software (Pharsight, Sunnyvale, CA, USA) was utilized to simulate the vancomycin concentration–time profiles for each patient. Subsequently, Matlab 7.0 software (MathWorks, Natick, MA, USA) was employed to calculate the AUC_24_ for each individual patient. The AUC_24_/MIC ratio was calculated based on the vancomycin MIC values of the strains.

### 4.5. Evaluation of Therapeutic Efficacy

Clinical success of vancomycin treatment was defined as disappearance of or improvement in clinical symptoms and signs of infection (such as fever and chills), return of laboratory results (such as white blood cell count and neutrophil count) to normal or pre-infection levels, and no need for vancomycin for at least 7 days after discontinuation. Clinical ineffectiveness was defined as the lack of improvement in infection-related symptoms and signs after vancomycin treatment or worsening of laboratory results. Specific auxiliary examinations, such as chest X-rays or CT scans indicating a reduction or disappearance of pulmonary lesions, are essential for evaluating clinical efficacy, especially in pneumonia cases.

The microbiological success of vancomycin treatment was defined as negative bacterial cultures after completing vancomycin treatment, or clinical success, even if microbiological examination specimens could not be repeated. Microbiological ineffectiveness was defined as positive bacterial cultures after completing vancomycin treatment, or clinical ineffectiveness, even if bacterial cultures could not be repeated.

### 4.6. Evaluation of Nephrotoxicity

Vancomycin nephrotoxicity indicates the occurrence of acute kidney injury (AKI). The definition of AKI, according to the KDIGO criteria [31], includes any of the following conditions (not graded): (a) an increase in serum creatinine (SCr) ≥26.5 μmol/L within 48 h; (b) a known or presumed increase in SCr 1.5 times the baseline value within the past 7 days; (c) urine output less than 0.5 mL/kg/h within 6 to 12 h.

### 4.7. Statistical Analysis

Statistical analysis was performed using IBM SPSS Statistics version 25.0 software (SAS Institute, Cary, NC, USA). Continuous variables were presented as median (interquartile range, IQR) and compared using an independent sample t-test or non-parametric tests. Categorical variables were expressed as number of cases (%) and compared using the chi-square test or Fisher’s exact test. Logistic regression analysis was used to calculate the odds ratio (OR) and corresponding 95% confidence interval (CI) for risk factors related to nephrotoxicity. Clinically significant variables with *p* < 0.1 in univariate regression were included in the multivariate logistic regression analysis, and adjusted odds ratios (aOR) were calculated. A significance level of *p* < 0.05 was considered statistically significant.

## 5. Conclusions

In conclusion, the incidence of vancomycin-associated nephrotoxicity was nearly three times higher in elderly patients than adult patients, but with similar clinical and microbiological efficacy. Risk factors for nephrotoxicity included C_trough_, AUC_24_, ICU admission, surgery, and drainage tube placement. Of these, a C_trough_ > 20 mg/L was an independent factor in elderly patients. In adult patients, vancomycin-associated nephrotoxicity was related to renal function and vancomycin levels. These findings suggest that renal function and vancomycin concentration need to be closely monitored in elderly patients to prevent nephrotoxicity.

## Figures and Tables

**Table 1:** Characteristics and outcomes of elderly versus adult patients.

Variables	Total (*n* = 164)	Elderly Patients (*n* = 83)	Adult Patients (*n* = 81)	*p* Value
**Demographics**				
Sex				
Male	114 (69.5)	53 (63.9)	61 (75.3)	0.111
Female	50 (30.5)	30 (36.1)	20 (24.7)	
Age (y)	66 (53, 80)	80 (75, 85)	53 (40, 60)	<0.001
BMI (kg/m^2^)	22.16 (19.97, 24.00)	22.00 (19.53, 24.00)	22.60 (20.01, 24.22)	0.324
**R** **enal function**				
Initial serum creatinine (µmol/L)	59.00 (46.00, 76.00)	64.00 (48.00, 95.50)	58.00 (44.50, 70.00)	0.181
Initial eGFR (mL/min/1.73 m^2^)	112.08 (85.41, 146.63)	102.01 (62.32, 140.23)	125.04 (97.66, 162.12)	0.002
Final serum creatinine (µmol/L)	56.00 (41.75, 80.25)	64.50 (44.75, 98.25)	51.00 (39.00, 65.00)	0.002
Final eGFR (mL/min/1.73 m^2^)	122.00 (80.07, 168.77)	94.27 (60.17, 127.88)	142.30 (111.21, 191.42)	<0.001
Change in serum creatinine (µmol/L)	2.00 (−7.50, 14.35)	8.00 (−6.00, 26.75)	−2.00 (−9.00, 7.00)	0.004
**Comorbidities**				
Cardiovascular disease	56 (34.1)	43 (51.8)	13 (16.0)	<0.001
Diabetes mellitus	27 (16.5)	22 (26.5)	5 (6.2)	<0.001
Stroke	53 (32.3)	36 (43.4)	17 (21.0)	0.002
COPD	9 (5.5)	9 (10.8)	0 (0.0)	0.003
Autoimmune disease	5 (3.0)	2 (2.4)	3 (3.7)	0.680
Trauma	17 (10.4)	7 (8.4)	10 (12.3)	0.411
Malignancy	30 (18.4)	13 (15.7)	17 (21.3)	0.357
Hematological malignancy	1 (0.6)	1 (1.2)	0 (0.0)	>0.999
Other diseases	37 (22.6)	20 (24.1)	17 (21.0)	0.634
**Exposures**				
Surgery	76 (46.3)	22 (26.5)	54 (66.7)	<0.001
Vascular catheter	110 (67.1)	53 (63.9)	57 (70.4)	0.375
Urinary catheter	94 (57.3)	45 (54.2)	49 (60.5)	0.416
Tracheal cannula	50 (30.5)	24 (28.9)	26 (32.1)	0.658
Tracheotomy	42 (25.6)	13 (15.7)	29 (35.8)	0.003
Drainage tube	41 (25.0)	14 (16.9)	27 (33.3)	0.015
ICU admission	92 (56.1)	45 (54.2)	47 (58.0)	0.623
ICU duration	26.50 (17.25, 40.00)	28.00 (18.50, 47.50)	26.00 (15.00, 35.00)	0.246
**Infection site**				
Bloodstream infection	20 (12.2)	8 (9.6)	12 (14.8)	0.311
Pneumonia	113 (68.9)	64 (77.1)	49 (60.5)	0.022
CNSI	4 (2.4)	1 (1.2)	3 (3.7)	0.364
SSTI	18 (11.0)	3 (3.6)	15 (18.5)	0.002
UTI	7 (4.3)	6 (7.2)	1 (1.2)	0.117
Other infection	14 (8.5)	5 (6.0)	9 (11.1)	0.244
**Concomitant antibiotics**				
β-lactams	93 (56.7)	45 (54.2)	48 (59.3)	0.515
Rifampicin	12 (7.3)	7 (8.4)	5 (6.2)	0.578
Quinolones	6 (3.7)	0 (0.0)	6 (7.4)	0.013
Others	39 (23.8)	19 (22.9)	20 (24.7)	0.787
**Vancomycin use parameters**				
Daily dose (g)	1.9 (1.2, 2.0)	1.4 (1.0, 2.0)	2.0 (1.8, 2.2)	<0.001
Duration (d)	12 (9, 17)	12 (9, 16)	13 (10, 18)	0.448
C_trough_ (mg/L)	10.88 (6.43, 15.92)	14.69 (10.58, 20.71)	7.82 (4.52, 12.57)	<0.001
C_peak_ (mg/L) ^a^	25.66 (19.59, 31.84)	26.63 (20.15, 32.94)	24.49 (19.04, 24.49)	0.112
AUC_24_ (mg·h/L) ^b^	404.19 (319.44, 547.20)	461.65 (353.01, 609.10)	387.56 (283.53, 462.09)	0.006
Vancomycin MIC ≥ 1 mg/L ^c^	90 (55.2)	41 (50.0)	49 (60.5)	0.178
AUC_24_/MIC ^b^	562.73 (363.38, 794.90)	647.97 (461.65, 913.42)	463.54 (330.09, 718.28)	0.007
**Outcome**				
Clinical success	112 (68.3)	56 (67.5)	56 (69.1)	0.819
Microbiological success	127 (77.4)	64 (77.1)	63 (77.8)	0.918
Nephrotoxicity	20 (12.2)	15 (18.1)	5 (6.2)	0.020

Data are presented as the median (interquartile range) or *n* (%). BMI: body mass index; eGFR: estimated glomerular filtration rate; COPD: chronic obstructive pulmonary disease; ICU: intensive care unit; CNSI: central nervous system infection; SSTI: skin and soft tissue infection; UTI: urinary tract infection. ^a^ Lack of vancomycin peak concentration in 7 elderly and 3 adult patients. ^b^ AUC_24_ and AUC_24_/MIC values could not be calculated for 7 elderly and 3 adult patients due to lack of peak concentration of vancomycin. ^c^ Lack of vancomycin MIC value in 1 elderly patient.

**Table 2:** Differences in clinical and vancomycin parameters between elderly and young adult patients classified according to nephrotoxicity.

Variables	Elderly Patients (*n* = 83)			Adult Patients (*n* = 81)		
	Non-Nephrotoxicity (*n* = 68)	Nephrotoxicity (*n* = 15)	*p* Value	Non-Nephrotoxicity (*n* = 76)	Nephrotoxicity (*n* = 5)	*p* Value
**Demographics**						
Sex						
Male	44 (64.7)	9 (60.0)	0.731	58 (76.3)	3 (60.0)	0.593
Female	24 (35.3)	6 (40.0)		18 (23.7)	2 (40.0)	
Age (y)	80 (74, 86)	79 (75, 84)	0.817	53 (39, 59)	46 (35, 63)	0.898
BMI (kg/m^2^)	21.48 (19.53, 23.83)	22.93 (19.67, 24.08)	0.516	22.16 (20.00, 24.22)	18.25 (8.32, 27.66)	0.331
**Renal function**						
Initial serum creatinine (µmol/L)	61.00 (44.00, 91.75)	70.60 (52.00, 106.00)	0.161	57.00 (43.00, 59.00)	121.00 (72.00, 353.50)	0.011
Initial eGFR (mL/min/1.73 m^2^)	104.48 (62.81, 143.68)	94.59 (45.70, 112.08)	0.113	127.72 (102.16, 172.91)	52.37 (22.38, 93.40)	0.003
Final serum creatinine (µmol/L)	56.80 (42.00, 79.00)	169.00 (102.00, 255.00)	<0.001	50.00 (39.00, 60.00)	259.40 (93.00, 425.50)	0.052
Final eGFR (mL/min/1.73 m^2^)	108.27 (81.74, 148.24)	34.77 (18.43, 60.65)	<0.001	145.41 (118.18, 194.04)	23.66 (11.98, 64.98)	<0.001
Change in serum creatinine (µmol/L)	2.80 (−8.00, 13.00)	98.00 (40.00, 220.00)	<0.001	−3.00 (−9.25, 5.25)	153.80 (18.50, 405.00)	<0.001
**Exposures**						
Surgery	14 (20.6)	8 (53.3)	0.020	52 (68.4)	2 (40.0)	0.327
Vascular catheter	42 (61.8)	11 (73.3)	0.399	54 (74.1)	3 (60.0)	0.630
Urinary catheter	35 (51.5)	10 (66.7)	0.285	47 (61.8)	2 (40.0)	0.379
Tracheal cannula	19 (27.9)	5 (33.3)	0.756	24 (31.6)	2 (40.0)	0.654
Tracheotomy	11 (16.2)	2 (13.3)	>0.999	27 (35.5)	2 (40.0)	>0.999
Drainage tube	8 (11.8)	6 (40.0)	0.017	25 (32.9)	2 (40.0)	>0.999
ICU admission	33 (48.5)	12 (80.0)	0.027	43 (56.6)	4 (80.0)	0.392
ICU duration	28.00 (18.25, 47.75)	27.00 (10.00, 43.00)	0.075	26.00 (12.00, 35.75)	24.00 (21.00, 25.50)	0.668
**Infection site**						
Bloodstream infection	5 (7.4)	3 (20.0)	0.153	11 (14.5)	1 (20.0)	0.561
Pneumonia	53 (77.9)	11 (73.3)	0.738	45 (59.2)	4 (80.0)	0.643
CNSI	1 (1.5)	0 (0.0)	>0.999	3 (3.9)	0 (0.0)	>0.999
SSTI	2 (2.9)	1 (6.7)	0.455	15 (19.7)	0 (0.0)	0.578
UTI	5 (7.4)	1 (6.7)	>0.999	1 (1.3)	0 (0.0)	>0.999
Other infection	5 (7.4)	0 (0.0)	0.579	9 (11.8)	0 (0.0)	>0.999
**Concomitant antibiotics**						
β-lactam	35 (51.5)	10 (66.7)	0.285	43 (56.6)	5 (100.0)	0.076
Rifampicin	5 (7.4)	2 (13.3)	0.605	5 (6.6)	0 (0.0)	>0.999
Other	15 (22.1)	4 (26.7)	0.738	18 (23.7)	2 (40.0)	0.593
**Vancomycin use parameters**						
Daily dose (g)	1.39 (1.00, 2.00)	1.50 (0.80, 2.00)	0.806	2.00 (1.90–2.20)	1.25 (0.66, 2.10)	0.068
Duration (d)	13.00 (10.00, 16.00)	10.00 (8.00, 13.00)	0.133	13.00 (10.00, 18.75)	10.00 (6.50, 10.00)	0.019
C_peak_ (mg/L) ^a^	25.96 (19.13, 31.95)	33.62 (26.40, 42.26)	0.012	24.01 (18.63, 29.78)	39.91 (27.67, 58.01)	0.009
C_trough_ (mg/L)	13.94 (9.96, 17.69)	22.65 (14.60, 26.13)	0.004	7.57 (4.41, 11.07)	25.93 (10.63, 43.45)	0.004
<10	17 (25.0)	2 (13.3)	0.502	55 (72.4)	1 (20.0)	0.030
10–15	23 (33.8)	2 (13.3)	0.212	13 (17.1)	1 (20.0)	>0.999
15–20	16 (23.5)	2 (13.3)	0.505	7 (9.2)	0 (0.0)	>0.999
>20	12 (17.6)	9 (60.0)	0.002	1 (1.3)	3 (60.0)	<0.001
AUC_24_ (mg·h/L) ^b^	450.01 (353.01, 561.77)	600.06 (340.53, 725.64)	0.060	367.32 (281.66, 456.82)	762.40 (664.80, 1022.70)	<0.001
<400	27 (42.9)	4 (33.3)	0.751	45 (60.8)	0 (0.0)	0.012
400–600	23 (36.5)	2 (16.7)	0.316	24 (32.4)	0 (0.0)	0.316
>600	13 (20.6)	6 (50.0)	0.064	5 (6.8)	5 (100.0)	<0.001
Vancomycin MIC ≥ 1 mg/L ^c^	33 (49.3)	8 (53.3)	0.775	48 (63.2)	1 (20.0)	0.077
AUC_24_/MIC ^b^	647.97 (442.72, 928.38)	681.81 (583.16, 859.99)	0.298	459.44 (328.73, 673.82)	1524.80 (1019.00, 2045.40)	0.001
<400	14 (22.2)	1 (8.3)	0.439	30 (40.5)	0 (0.0)	0.151
400–600	16 (25.4)	4 (33.3)	0.723	22 (29.7)	0 (0.0)	0.315
>600	33 (52.4)	7 (58.3)	0.705	22 (29.7)	5 (100.0)	0.004

Data are presented as the median (interquartile range) or *n* (%). BMI: body mass index; eGFR: estimated glomerular filtration rate; ICU: intensive care unit; CNSI: central nervous system infection; SSTI: skin and soft tissue infection; UTI: urinary tract infection. ^a^ Lack of vancomycin peak concentration in 7 elderly and 3 adult patients. ^b^ AUC_24_ and AUC_24_/MIC values could not be calculated for 7 elderly and 3 adult patients due to lack of peak concentration of vancomycin. ^c^ Lack of vancomycin MIC value in 1 elderly patient.

**Table 3:** Multivariate analysis of risk factors for vancomycin-associated nephrotoxicity in elderly patients.

Characteristics	Univariate Analysis		Multivariate Logistic Analysis	
	OR (95% CI)	*p* Value	aOR (95% CI)	*p* Value
Surgery	4.408 (1.365–14.237)	0.013	3.933 (0.821–18.839)	0.087
Drainage tube	5.000 (1.405–17.793)	0.013	1.838 (0.185–18.218)	0.603
ICU admission	4.242 (1.098–16.391)	0.036	4.177 (0.695–25.089)	0.118
C_peak_ (mg/L)	1.035 (0.997–1.075)	0.072	1.003 (0.944–1.066)	0.913
C_trough_ (mg/L)	1.132 (1.044–1.228)	0.003		
Reference, <10	0.462 (0.094–2.256)	0.340	-	-
10–15 (exclude 15)	0.301 (0.063–1.449)	0.134	0.520 (0.052–5.153)	0.576
15–20	0.500 (0.102–2.453)	0.393	0.510 (0.047–5.491)	0.579
>20	7.000 (2.095–23.394)	0.002	8.936 (1.943–41.095)	0.005
AUC_24_ (mg·h/L)	1.003 (1.000–1.006)	0.049		
Reference, <400	0.667 (0.182–2.446)	0.541	-	-
400–600	0.348 (0.070–1.727)	0.196	0.374 (0.049–2.873)	0.344
>600	3.500 (0.975–12.563)	0.055	0.583 (0.028–11.958)	0.726

OR: odds ratio; aOR: adjusted odds ratio; CI: confidence interval; ICU: intensive care unit.

**Table 4:** Characteristics of elderly versus adult patients in subgroup of patients with eGFR > 60 mL/min/1.73 m^2.^

Variables	Elderly Patients (*n* = 65)	Adult Patients (*n* = 75)	*p* Value
**Demographics**			
Sex			
Male	42 (64.6)	56 (74.7)	0.196
Female	23 (35.4)	19 (25.3)	
Age (y)	80 (74, 86)	53 (39, 59)	<0.001
BMI (kg/m^2^)	21.97 (19.54, 23.44)	22.09 (20.00, 24.21)	0.373
**Renal function**			
Initial serum creatinine (µmol/L)	53.00 (40.50, 68.00)	56.00 (43.00, 69.00)	0.756
Initial eGFR (mL/min/1.73 m^2^)	111.67 (95.34, 150.13)	127.72 (103.64, 172.91)	0.076
Final serum creatinine (µmol/L)	56.80 (42.00, 79.50)	50.00 (39.00, 62.25)	0.016
Final eGFR (mL/min/1.73 m^2^)	108.72 (81.79, 150.33)	144.80 (117.86, 195.22)	<0.001
Change in serum creatinine (µmol/L)	9.00 (−3.00, 22.65)	−2.50 (−9.00, 6.25)	<0.001
**Exposures**			
Surgery	16 (24.6)	51 (68.0)	<0.001
Vascular catheter	41 (63.1)	54 (72.0)	0.260
Urinary catheter	37 (56.9)	46 (61.3)	0.596
Tracheal cannula	18 (27.7)	24 (32.0)	0.579
Tracheotomy	10 (15.4)	28 (37.3)	0.004
Drainage tube	10 (15.4)	26 (34.7)	0.009
ICU admission	33 (50.8)	32 (42.7)	0.397
ICU duration			
**Infection site**			
Bloodstream infection	7 (10.8)	11 (14.7)	0.492
Pneumonia	49 (75.4)	44 (58.7)	0.037
CNSI	1 (1.5)	3 (4.0)	0.623
SSTI	3 (4.6)	15 (20.0)	0.007
UTI	5 (7.7)	1 (1.3)	0.096
Other infection	4 (6.2)	9 (12.0)	0.235
**Concomitant antibiotics**			
β-lactam	35 (53.8)	42 (56.0)	0.798
Rifampicin	6 (9.2)	5 (6.7)	0.574
Quinolones	0 (0.0)	5 (6.7)	0.034
Other	15 (23.1)	18 (24.0)	0.898
**Vancomycin use parameters**			
Daily dose (g)	1.50 (1.00, 2.00)	2.0 (1.91, 2.20)	<0.001
Duration (d)	12 (10, 16.5)	13 (10, 18)	0.518
C_trough_ (mg/L)	13.87 (9.83, 20.16)	7.50 (4.38, 11.36)	<0.001
C_peak_ (mg/L) ^a^	26.38 (19.43, 32.89)	24.12 (18.46, 29.43)	0.112
AUC_24_ (mg·h/L) ^b^	450.01 (353.01, 587.96)	359.14 (281.49, 457.33)	0.004
Vancomycin MIC ≥ 1 mg/L ^c^	30 (46.9)	48 (64.0)	0.043
AUC_24_/MIC ^b^	653.88 (467.90, 900.02)	458.33 (328.12, 668.66)	0.001
**Outcome**			
Clinical success	45 (69.2)	51 (68.0)	0.876
Microbiological success	50 (76.9)	58 (77.3)	0.954
Total success	43 (66.2)	49 (65.3)	0.919
Nephrotoxicity	10 (15.4)	2 (2.7)	0.007

Data are presented as the median (interquartile range) or *n* (%). BMI: body mass index; eGFR: estimated glomerular filtration rate; ICU: intensive care unit; CNSI: central nervous system infection; SSTI: skin and soft tissue infection; UTI: urinary tract infection. ^a^ Lack of vancomycin peak concentration in 6 elderly and 2 adult patients. ^b^ AUC_24_ and AUC_24_/MIC values could not be calculated for 6 elderly and 2 adult patients due to lack of peak concentration of vancomycin. ^c^ Lack of vancomycin MIC value in 1 elderly patient.

## Data Availability

The data presented in this study are available on request from the corresponding author.

## Supplementary Materials

The following supporting information can be downloaded at: https://www.mdpi.com/article/10.3390/antibiotics13040324/s1, Figure S1. Comparison of C_trough_, AUC_24_, and AUC_24_/MIC in elderly and adult patients; Figure S2. Distribution of vancomycin MIC value in MRSA of elderly and adult patients; Figure S3: ROC analysis of vancomycin and C_trough_ in elderly patients; Figure S4. Correlation of C_trough_ and AUC_24_ of elderly and adult patients; Table S1: Multivariate analysis of risk factors for nephrotoxicity in subgroup of adult patients.
