# Supplementary material for: Age-Related Differences in Vancomycin-Associated Nephrotoxicity and Efficacy in Methicillin-Resistant Staphylococcus aureus Infection: A Comparative Study between Elderly and Adult Patients
Source: Antibiotics (Basel). 2024 Apr 3;13(4):324. doi: 10.3390/antibiotics13040324 (PMC11047698; doi:10.3390/antibiotics13040324)
Supplement: Supplementary file 1 [file antibiotics-13-00324-s001.zip › Figure S3.pdf]

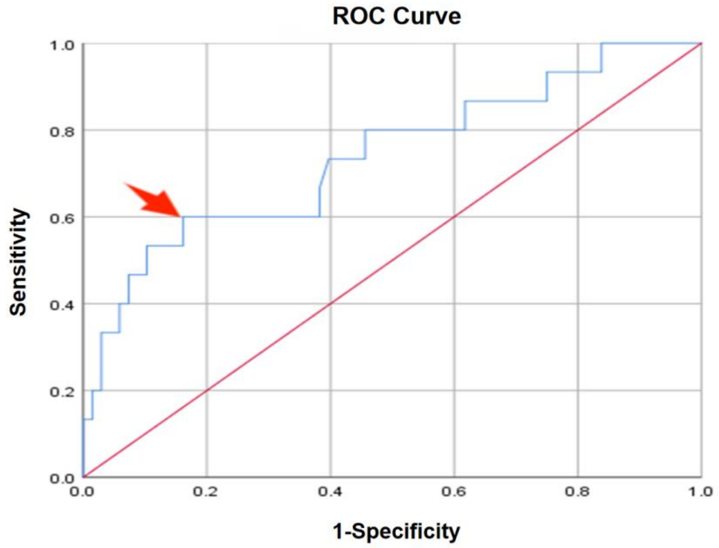

Figure S3. ROC analysis of vancomycin and C_{trough} in elderly patients. Red arrow indicates the optimal cutoff. The optimal cutoff value was 20.78 mg/L, with a sensitivity of 60.0% and a specificity of 83.8%, respectively.
