# Supplementary material for: Age-Related Differences in Vancomycin-Associated Nephrotoxicity and Efficacy in Methicillin-Resistant Staphylococcus aureus Infection: A Comparative Study between Elderly and Adult Patients
Source: Antibiotics (Basel). 2024 Apr 3;13(4):324. doi: 10.3390/antibiotics13040324 (PMC11047698; doi:10.3390/antibiotics13040324)
Supplement: Supplementary file 1 [file antibiotics-13-00324-s001.zip › Table S1.pdf]

Table S1. Multivariate analysis of risk factors for nephrotoxicity in subgroup of adult patients

Characteristics	Univariate analysis		Multivariate analysis
	OR (95% CI)	*P* Value	*P* Value
Initial serum creatinine (μmol/L)	1.012 (1.002-1.022)	0.019	0.974
Initial eGFR (mL/min/1.73m^2)	0.959 (0.933-0.986)	0.004	0.975
Daily dose (g)	0.117 (0.022-0.627)	0.012	0.981
C_{trough} (mg/L)	1.253 (1.067-1.471)	0.006	0.976
C_{peak} (mg/L)	1.109 (1.032-1.191)	0.005	0.989
AUC_{24} (mg·h/L)	1.014 (1.004-1.024)	0.006	0.975
AUC_{24}/MIC	1.004 (1.002-1.007)	0.002	0.989

OR: odds ratio; CI:confidence interval
