# Supplementary figures and images for: Age-Related Differences in Vancomycin-Associated Nephrotoxicity and Efficacy in Methicillin-Resistant Staphylococcus aureus Infection: A Comparative Study between Elderly and Adult Patients
Source: Antibiotics (Basel). 2024 Apr 3;13(4):324. doi: 10.3390/antibiotics13040324 (PMC11047698; doi:10.3390/antibiotics13040324)

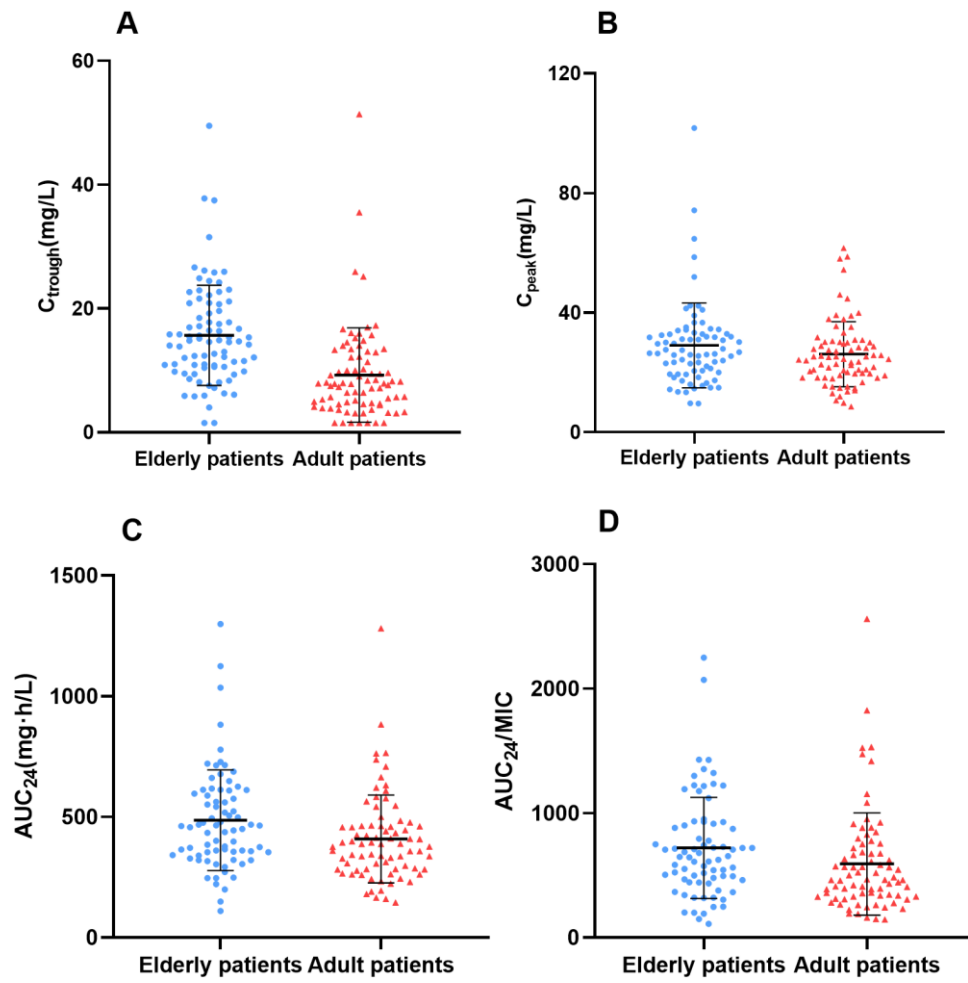

Figure S1. Comparison of  $C_{trough}$  (A),  $C_{trough}$  (B),  $AUC_{24}$  (C) and  $AUC_{24}/MIC$  (D) in elderly and adult patients.

Supplement: Supplementary file 1 [file antibiotics-13-00324-s001.zip › Figure S1.pdf]

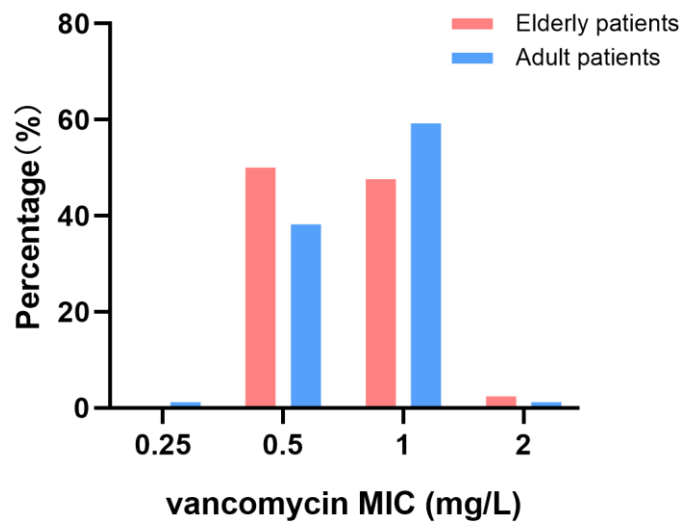

Figure S2. Distribution of vancomycin MIC value in MRSA of elderly and adult patients

Supplement: Supplementary file 1 [file antibiotics-13-00324-s001.zip › Figure S2.pdf]

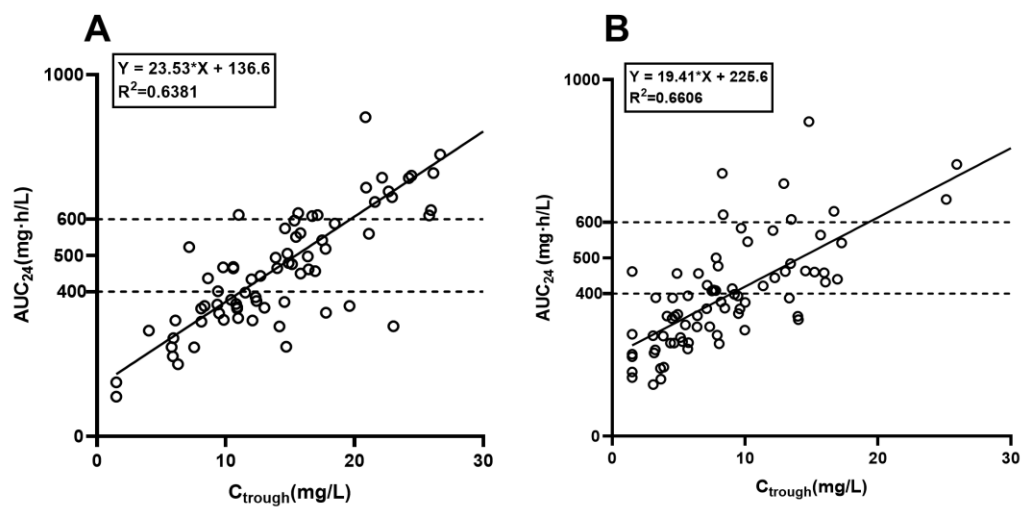

Figure S4. Correlation of  $C_{trough}$  and  $AUC_{24}$  of elderly (A) and adult (B) patients

Supplement: Supplementary file 1 [file antibiotics-13-00324-s001.zip › Figure S4.pdf]
